# Thermal Limits and Decline of *Synechococcus* Under Accelerated Warming and Marine Heatwaves

**DOI:** 10.1111/gcb.70791

**Published:** 2026-03-11

**Authors:** Luthfiyyah Azizah, Eva Alou‐Font, Alexandra Coello‐Camba, Susana Agusti

**Affiliations:** ^1^ Biological and Environmental Science and Engineering Division King Abdullah University of Science and Technology (KAUST) Thuwal Saudi Arabia; ^2^ Universidad Internacional de Valencia (VIU) Valencia Spain

**Keywords:** accelerated warming, heatwaves, high‐frequency sampling, phenology, picophytoplankton, Red Sea, *Synechococcus*, thermal limits, tropical ocean

## Abstract

Marine picophytoplankton contribute roughly 20% of global oceanic primary production, including thermally resilient taxa such as *Synechococcus*, which dominate warm oceans and are projected to benefit from future warming. Tropical populations exist near their upper thermal limits, making them highly vulnerable to further warming, a largely unexplored risk for *Synechococcus*. Here, we combine high‐frequency in situ observations and laboratory experiments to examine the thermal tolerance of *Synechococcus* in the Red Sea, one of the warmest marine basins globally. Over a 7‐year period (2018–2024), we monitored population dynamics alongside continuous sea surface temperatures, capturing the increasing frequency and duration of marine heatwaves (MHWs) in 2023–2024, the warmest years on record. Abundance of *Synechococcus* increased with temperature and peaked at ~30.2°C, but extreme temperatures recorded in 2023–2024 substantially exceeded the range associated with maximum *Synechococcus* abundance. Laboratory experiments of *Synechococcus* clades isolated from the Red Sea, confirmed strain‐specific optima ranging from ~25°C (clade IIIa) to ~33°C (clade IIa), with maximum thermal limit up to 35.2°C. During the unprecedented warming of 2023–2024, when sea surface temperatures exceeded 35°C and MHWs persisted for up to 55 days, *Synechococcus* blooms weakened by ~4.5‐fold. Although the timing of *Synechococcus* blooms remained stable, warmer years were characterized by reduced abundances and lower bloom magnitude, indicating changes in population intensity rather than shifts in bloom timing. Comparison with published temperature–abundance models demonstrates prior datasets fail to capture responses to extreme warming. Our results provide direct evidence of ecological niche loss in tropical *Synechococcus*, challenging predictions of their future dominance and highlighting the vulnerability of even the most heat‐tolerant primary producers to accelerated warming. These findings underscore the capacity of extreme warming events to rapidly destabilize plankton communities, reduce primary production, and alter ecosystem function, emphasizing increasing uncertainty in forecasting ocean productivity under accelerating climate change.

## Graphical abstract

Marine picophytoplankton, including *Synechococcus*, contribute ~20% of ocean primary production and are considered thermally resilient. However, 7 years of observations and experiments in the Red Sea show that recent extreme warming reduced *Synechococcus* abundance. Seawater temperatures during marine heatwaves in 2023–2024 exceeded the thermal limits of clade IIa, the most thermally tolerant, weakening blooms and revealing an unexpected vulnerability of heat‐tolerant picophytoplankton to accelerated climate change. These findings challenge current assumptions about future ocean productivity and marine ecosystem resilience.

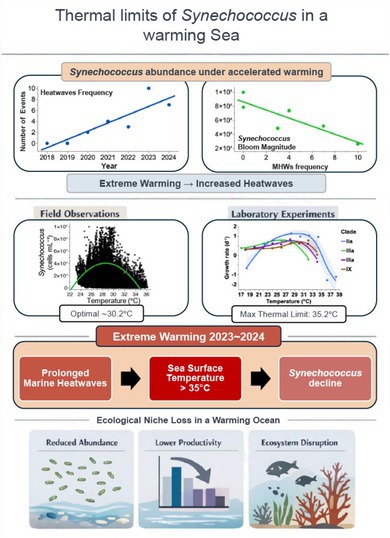

## 1 Introduction

Ocean warming presents substantial challenges for marine organisms and ecosystems, with increasing evidence of its effects on species distribution and migration, food webs, and phenology (Edwards and Richardson 2004; Jorda et al. 2020; O'Connor et al. 2009; Poloczanska et al. 2013; Cabrerizo et al. 2024). These changes have far‐reaching consequences on marine life and human societies depending on these ecosystems (Allison and Bassett 2015; Gattuso et al. 2015). The World Meteorological Organization confirmed that 2023 and 2024 have been the warmest years on record, with global temperatures 1.45°C and 1.55°C above the preindustrial average, respectively (Nullis 2025; WMO 2024). NASA also reported that the summer of 2024 was about 0.1°C warmer than any other summer in NASA records (Younger 2024). The impacts of warming are particularly severe during marine heatwaves (MHWs), which can cause large‐scale disruptions in the marine ecosystems, such as coral bleaching (Hughes et al. 2017), mass mortality of seagrass meadows (Nguyen et al. 2021; Marbà et al. 2022), and declines in fish stocks (Pörtner and Peck 2010; Cheung et al. 2021). The severity of these impacts varies by trophic level and biome, with higher trophic levels frequently experiencing major disruptions (de Luzinais et al. 2024). However, small organisms with fundamental ecological roles as phytoplankton, may also be highly vulnerable to warming, with potentially far‐reaching global consequences. Several studies have reported declines in oceanic primary production under warming conditions (Behrenfeld et al. 2006; Ryan‐Keogh et al. 2025) and shifts in photosynthetic plankton communities toward smaller sizes (Sommer and Lengfellner 2008; Morán et al. 2010). These changes can ultimately impact highher trophic levels (Kiørboe 1993; Daufresne et al. 2009) and disrupt global biogeochemical cycles (Mackenzie et al. 2000; Hayashida et al. 2020).

Marine picophytoplankton (diameter: < 3 μm) contribute approximately 20% of global oceanic primary production (Uitz et al. 2010) and dominate biomass and production (> 60%) in tropical and subtropical oceans (Agawin et al. 2000; Buitenhuis et al. 2012). *Synechococcus*, a key component of picophytoplankton, plays a crucial role in marine ecosystems as one of the most abundant photosynthetic organisms in the global ocean (Waterbury et al. 1979; Dvořák et al. 2014; Scanlan and West 2002). It inhabits diverse environments, from coastal to open oceans, and is found globally, with predominant abundance in subtropical and tropical waters (Li 1998; Agustí et al. 2019). *Synechococcus* is known for its high thermal tolerance, as indicated by its response to increasing seawater temperatures (Agawin et al. 1998; Morán et al. 2010; Stevens et al. 2023), confirmed by its wide genetic diversity (Doré et al. 2022). Observations from temperate regions report an increasing abundance of *Synechococcus* in recent decades (Hunter‐Cevera et al. 2016; Morán et al. 2010; Schmidt et al. 2020), predicting that its dominance will continually grow in a warming ocean (Flombaum et al. 2013; Brander and Kiørboe 2020). Thriving in warm, oligotrophic waters, *Synechococcus* is highly dominant in the tropical Red Sea (Al‐Otaibi et al. 2020; Coello‐Camba and Agustí 2021, 2024), one of the warmest marine environments on Earth, where warming exceeds the global average (Chaidez et al. 2017). Tropical ecosystems are identified among those most vulnerable to rising temperatures and are expected to experience the greatest biodiversity loss (Thomas et al. 2012; Tittensor et al. 2010). Tropical organisms are particularly sensitive to temperature changes, as many already inhabit environments near their thermal limits (Deutsch et al. 2008; Thomas et al. 2012). As ocean temperatures rise, they face the unprecedented challenge of experiencing temperatures beyond their historical range, leading to habitat loss and potential ecological shifts.

Herein, we report that extreme seawater temperatures observed over the past 2 years in a tropical sea have exceeded the thermal tolerance of warm‐adapted *Synechococcus* populations. Over the past 7 years, we continuously monitored the population dynamics of *Synechococcus* in the Red Sea—one of the warmest marine environments—using a high‐frequency automatic sampling system. This unprecedented dataset captured the rising frequency and duration of marine heatwaves (MHWs) in 2023–2024, during which seawater temperatures reached a record 36.63°C. These observations provide valuable insights into how warming affects these microorganisms. As expected from literature data, *Synechococcus* abundance increased with rising temperatures. However, the unprecedented warming in recent years has pushed seawater temperature well beyond their thermal optimum, causing a marked decline in populations, reducing bloom magnitude and a decrease in annual abundance linearly related to the increasing number and duration of heatwaves.

## 2 Materials and Methods

### 2.1 Sampling and Study Site

High‐resolution monitoring began in January 2018 and continued through December 2024 at the Ibn Sina Field Research Station (22° 20.531′ N, 39° 5.219′ E) at the Red Sea coast, a pristine area surrounded by coral reefs. Plankton subsurface sampling was performed hourly using a remotely controlled automated underwater flow cytometer (CytoSub, CytoBuoy b.v., the Netherlands) mounted on a buoy. The system was connected to a land‐based power source and the internet through an underwater cable, enabling real‐time data transmission to a laboratory computer. The CytoSub was programmed for hourly sampling with settings optimized for picoplankton communities. During equipment maintenance, discrete water samples were collected near the buoy at varying frequencies, from daily to weekly; this included intermittent sampling in 2018 and 2020. Fresh samples were analyzed immediately using a benchtop flow cytometer (CytoSense, CytoBuoy b.v., the Netherlands). In 2024, the sampling frequency was reduced to four daily acquisitions in May–December. Data collection was interrupted from March to September 2020 because of the COVID‐19 restrictions. Additional sampling using the CytoBuoy flow cytometer was conducted in December 2017 and January 2025 to define the start and end of blooms. Sea surface temperature (SST) and salinity were recorded with a CTD (Ocean Seven 310 Plus, Idronaut) attached to the mooring frame housing the CytoSub. Measurements were taken every 30 min throughout the study. The CTD was replaced monthly to ensure continuous data collection. During periods of CTD maintenance or malfunction (2022), temperature was recorded using a HOBO Pendant Temperature Data Logger (UA‐001‐64, Onset Computer Corporation, Bourne, MA, USA). Prior to deployment, HOBO UA‐001‐64 temperature loggers were calibrated under stable laboratory conditions (~22.5°C) for 12 h. We used remotely sensed SST data from Copernicus Marine Service OSTIA (GHRSST framework, UK Met Office) to fill in situ temperature data gaps.

### 2.2 Characterization of Environmental Parameters

Surface water samples were collected biweekly from a depth of 1 m around the buoy to measure additional environmental parameters. Nutrient concentrations were analyzed for 2021–2023, and the first 5 months of 2024. For each sample, 15 mL of seawater was transferred into acid‐cleaned polyethylene flasks and stored at −20°C until analysis. ${\mathrm{NO}}_{3}^{-}$, ${\mathrm{NO}}_{2}^{-}$, ${\mathrm{SiO}}_{2}^{4-}$, and ${\mathrm{PO}}_{4}^{3-}$ concentrations were analyzed using a Segmented Flow Analyzer (SEAL Analytical) following standard autoanalyzer techniques (Hansen and Koroleff 1999).

### 2.3 High‐Frequency Underwater Sampling of *Synechococcus* Populations

The CytoSub and CytoSense (CytoBuoy b.v., the Netherlands) flow cytometers are provided with a 488 nm solid state laser exciting photosynthetic pigments. Photomultiplier tubes detect forward scatter (FSC), side scatter (SSC), and are able to detect particles ranging from 0.2 μm to 4 mm. Additionally, an integrated camera captures high‐resolution images of individual cells upon detection, linking morphological features to optical data. In situ collection of samples was configured using the FL‐Red channel (25 mV threshold) as the trigger and maintained a sample pump speed of 1.36 μL s^−1^ for a maximum duration of 2 min. The sampling protocol was optimized for *Synechococcus*, characterized by low FSC and SSC signals, low red fluorescence, and high orange fluorescence.

Before deployment, the CytoBuoy was calibrated using a known concentration of ±10^3^ beads/mL^−1^ (3 μm beads, Sysmex) to ensure accurate particle counts. Size calibration was conducted using 1 μm beads provided by the manufacturer. We performed regular maintenance every ±3 months, including tube cleaning, replacing filtered seawater in the cartridge, and adding biocide before redeployment. As water flowed through the system, phytoplankton cells were detected in real time, with FSC, SSC, and fluorescence signals (chlorophyll *a*, phycocyanin, and phycoerythrin) recorded alongside high‐resolution images.

Data were analyzed using CytoClus (CytoBuoy b.v., the Netherlands) and EasyClus (Thomas Rutten projects) software to quantify *Synechococcus* abundance (cells mL^−1^) and size (length, μm). Cytograms used for identification included total FSC versus total red fluorescence, total SSC versus total orange fluorescence, and total orange fluorescence versus total red fluorescence. Owing to its small size (< 3 μm) and strong orange fluorescence, *Synechococcus* was distinctly identified and marked as blue dots in the cytograms in (Figure S1).

### 2.4 Global Patterns of *Synechococcus* Abundance in Relation to Temperature

We examined published studies describing the relationship between *Synechococcus* abundance and temperature across a broad range of marine environments, spanning temperate, subtropical, and tropical regions. For temperate waters (0.6°C–30°C), we compiled the published abundance–temperature relationships and fitted equations reported for the Chesapeake Bay (Wang et al. 2011), the North Atlantic Ocean (Stevens et al. 2023; Morán et al. 2010), and the East Sea (the East China Sea and Sea of Japan) (Choi et al. 2013). Relationships from subtropical regions (12.4°C–30.5°C) were represented by reported model fits from studies conducted in the South China Sea (Chen et al. 2011), the coast of Taiwan (Chang et al. 1996), and the Subtropical Atlantic, Indian, and Pacific Oceans (Agustí et al. 2019). Additionally, we included data from the Red Sea open waters (21.4°C–32.4°C) (Coello‐Camba and Agustí 2021).

These analyses are based on equations and functional relationships reported in the original studies, and not on re‐analysis of the original datasets. For studies that did not provide explicit mathematical relationships, we extracted data points from the linear and Lorentzian fits described in the published plots using WebPlotDigitizer and applied linear or Lorentzian fits in accordance with the publications. When provided, we reproduced the linear or Lorentzian fits. In this sense, we reproduce the linear fit provided in Agustí et al. (2019) from tropical and subtropical data, which included the equation:

(1) $$\log\;\text{Synechococcus}\;\text{abundance}\;\left(\text{cells}\;{\mathrm{mL}}^{-1}\right)=2.55+0.032\left(T\right)$$

and the Lorentzian fit equation from Red Sea open waters provided in Coello‐Camba and Agustí (2021):

(2) $$\log\;\text{Synechococcus}\;\text{abundance}\;\left(\text{cells}\;{\mathrm{mL}}^{-1}\right)=4/\left(1+{\left(\frac{T-30.6}{8}\right)}^{2}\right)$$

where *T* indicates temperature (°C) in both equations.

### 2.5 *Synechococcus* Phenology

We explored temporal variations in *Synechococcus* abundance to assess changes in bloom phenology, including the timing of bloom initiation, duration, peak, and termination over the study period. Bloom initiation was defined as the first week in which abundance exhibited a sustained positive slope for at least 5 consecutive days. The initial growth phase was defined as the period immediately following bloom initiation during which cell abundance consistently increased prior to reaching peak abundance. Additionally, we analyzed seasonal patterns by calculating the monthly average *Synechococcus* abundance. In 2020, due to extensive data gaps, interpolation was not applied.

### 2.6 *Synechococcus* Thermal Performance

We analyzed the thermal tolerance of *Synechococcus* strains from the Red Sea by testing representatives of the local genetic pool, including subclades IIIa, IIa, and IX. The strains used herein were obtained from the Roscoff Culture Collection (RCC, http://roscoff‐culture‐collection.org) and originally isolated from the Gulf of Aqaba (Red Sea): RS9905 (RCC 2372, subclade IIIa), RS9915 (RCC 2553, subclade IIIa), RS9912 (RCC 2384, subclade IIa), and RS9901 (RCC 2529, subclade IX). Once in our laboratory, the strains were maintained at the same temperature as in the RCC (22°C), using a similar PCR‐S11 medium prepared with 0.2 μm filtered and autoclaved Red Sea water, thereby preserving Red Sea salinity conditions, and under an irradiance of ~30 W m^−2^. For the temperature performance experiments, replicated cultures (*n* = 3–4) were grown in similar PCR‐S11 medium and conditions exposed to temperature gradients in 2°C–3°C increments, spanning a range from 17°C to 38°C. The cultures were incubated in Percival chambers (Model I‐22LLVL, Geneva Scientific) under a 12 h/12 h light/dark cycle with a light intensity of 31.8 Wm^−2^ (~145.5 μmol photons m^−2^ s^−1^). Samples were collected daily or every 2 days to measure cell abundance using flow cytometry (CyFlow Space, Sysmex Co., or CytoSense, CytoBuoy b.v., the Netherlands) throughout the exponential and stationary growth phases. When no growth was observed, monitoring continued for an additional 2–3 weeks to detect potential delayed responses.

Growth rates (*h*(*T*), day^−1^) for each strain were calculated as the slope of the natural logarithm (Ln) of cell abundance over time (days) during the exponential phase. Thermal performance curves were generated by plotting growth rates (*h*(*T*)) across temperature treatments. The optimum growth temperature (*T*_opt_) was determined as the temperature at which maximum growth occurred using the unimodal function described by Dell et al. (2011):

(3) $$h\left(T\right)=\frac{{\mathrm{ce}}^{-\frac{E}{\mathrm{kT}}}}{1+e^{-\frac{1}{\mathrm{kT}}\left(E_{D}-\left(\frac{E_{D}}{T_{\mathrm{opt}}}+k\;\ln\left(\frac{E}{E_{D}-E}\right)\right)T\right)}}$$

Here, the thermodynamic parameter *E*_D_ determines the rate of decline in growth at temperatures exceeding *T*_opt_, while *c* is a constant. All unimodal responses were fitted to this model using nonlinear least‐squares regression. We applied the Gauss–Newton algorithm with a stopping limit of 1000 iterations. This method requires a series of initial values for each parameter to work. Herein, we assigned these values arbitrarily such that they were low enough to ensure proper algorithm performance, full data coverage, improved convergence, and accurate parameter estimation. We estimated the critical thermal minimum (*T*_min_) and maximum (*T*_max_) as the lowest and highest temperatures, respectively, at which no growth occurs.

### 2.7 Marine Heatwaves

We examined marine heatwave (MHW) events over the 7‐year study period, identifying them as periods when SST exceeded the 90th percentile of the 7‐year average for > 5 consecutive days (Hobday et al. 2016; Sen Gupta et al. 2020). MHW frequency was calculated as the number of events per year, whereas MHW duration was measured in days. The total MHW days per year were determined by summing the durations of all events within that year. Additionally, we quantified heat dissipation per month by calculating the slope between the maximum temperature in summer and the temperature observed at bloom initiation.

### 2.8 Statistical Analyses

Interannual variations in *Synechococcus* abundance, SST, and nutrient concentrations were analyzed using ANOVA, and a Tukey–Kramer post hoc test was applied following ANOVA to identify which specific group means differed significantly; significance level was set at *p* < 0.05. *Synechococcus* abundance data were log‐transformed prior to analysis to normalize the distribution and reduce heteroscedasticity, and linear and Lorentzian regression fits were applied to assess relationships with temperature. Linear regression was used to describe the first‐order temperature–abundance trends, while the Lorentzian model was applied to explore non‐linear curvature; both approaches were included to facilitate comparison with relationships reported in the literature. To determine the optimum growth temperature, we fitted a Gaussian model to the relationship between absolute values of *Synechococcus* abundance and temperature, allowing clearer identification of symmetry around the thermal optimum. Correlation and linear regressions were also used to examine trends in environmental parameters, MHWs and their relationship with *Synechococcus* abundance and other parameters. Residuals were examined to assess linearity, normality, homoscedasticity, and independence of errors. All results are reported as means ± standard error. Statistical analyses were conducted using JMP Pro 18 software.

## 3 Results

### 3.1 High‐Frequency Sampling in the Warmest Sea

Our time series, from January 2018 to December 2024, included quantification of *Synechococcus* abundance and recordings of sea surface temperatures (SSTs) (Figure S2) from the coastal station. *Synechococcus* was present throughout the study period, with an average abundance of 1.26 × 10^4^ ± 1.06 × 10^2^ cells mL^−1^, ranging from 7.14 × 10^1^ to 9.97 × 10^4^ cells mL^−1^ (Table 1). SSTs remained high, fluctuating between 22.58°C in February 2020 and 36.63°C in August 2023, with a similarly high temperature of 36.62°C in August 2024. The coldest years were 2018 and 2019, whereas the warmest years were 2023 and 2024, which is consistent with global trends (Table 1). We observed significant variations in *Synechococcus* abundance across the years. The lowest mean annual abundance occurred in 2023 (*p* < 0.0001), whereas the highest was recorded in 2018 and 2019 (the coldest years) (Table 1). Inorganic nutrient concentrations remained low throughout the study, which is consistent with oligotrophic conditions, although higher values were observed in 2023 (Figure S3), no significant variations were observed between years (Table S1). Moreover, silicate, nitrate, and phosphate levels showed slight seasonal variation. Nitrate and phosphate concentrations increased in January and between May and July, although these differences were not statistically significant (Figure S3). Salinity averaged 39.45 ± 0.005 PSU (*N* = 28,581) and was significant and positively correlated with SST (𝜌 = 0.41, *p* < 0.0001), whereas inorganic nutrients were not correlated with either salinity or SST.

**TABLE 1:** Annual changes in *Synechococcus* abundance and surface seawater temperature (SST) (2018–2024). Yearly averages (±SE), minimum and maximum values, and number of observations for *Synechococcus* abundance and SST are shown. Differences in sample size (*N*) reflect variations in sampling frequency. Years sharing the same letter are not significantly different (*p* < 0.05, ANOVA, Tukey's HSD test).

Year	*Synechococcus* abundance (cells mL^−1^)				SST (°C)			
	*N*	Mean (×10^3^) ± SE	Min	Max (×10^3^)	*N*	Mean ± SE	Min	Max
2018	109	24.0^A^ ± 2.09	1140	99.7	511	28.47^A^ ± 0.08	23.90	33.20
2019	2444	24.7^A^ ± 0.51	94.1	99.1	2070	27.94^B^ ± 0.05	23.85	32.44
2020	1723	11.5^B^ ± 0.25	147	82.8	2719	29.61^C^ ± 0.08	22.58	35.95
2021	4687	17.0^C^ ± 0.24	280	99	5270	31.30^D^ ± 0.03	24.90	35.82
2022	3479	10.1^D^ ± 0.19	84.2	75.4	7787	30.38^E^ ± 0.03	23.35	35.97
2023	5800	6.9^E^ ± 0.10	85.8	74.5	8689	30.24^F^ ± 0.03	23.98	36.63
2024	2492	9.2^D^ ± 0.22	71.4	86.3	8450	30.29^EF^ ± 0.03	24.52	36.62

### 3.2 Decline in *Synechococcus* Population

**FIGURE 1:**
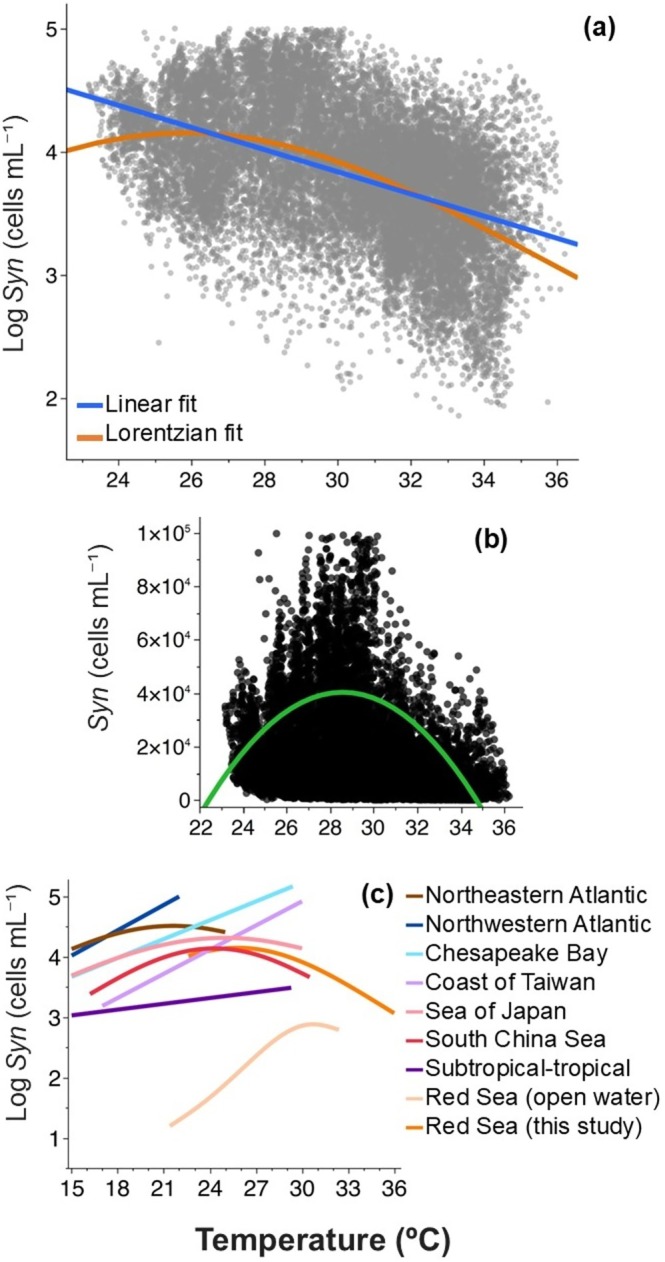
Relationship between *Synechococcus* abundance and temperature. (a) High‐frequency abundance data of *Synechococcus* in the coastal Red Sea (2018–2024) showing the relationship with temperature. Red line: Linear fit (*R* ^2^ = 0.223, *p* < 0.0001); blue line: Lorentzian fit (*R* ^2^ = 0.244, *p* < 0.0001). (b) Gaussian model identifying the temperature threshold (30.23°C) for maximum *Synechococcus* abundance. (c) Literature‐based relationships between temperature and *Synechococcus* abundance from different marine regions: Northeastern Atlantic (brown line) (Stevens et al. 2023), Northwestern Atlantic (dark blue line) (Morán et al. 2010), Chesapeake Bay (light blue line) (Wang et al. 2011), Coast of Taiwan (light purple line) (Chang et al. 1996), Sea of Japan (pink line) (Choi et al. 2013), South China Sea (red line) (Chen et al. 2011), Subtropical–Tropical Atlantic, Indian, and Pacific Oceans (dark purple line) (Agustí et al. 2019), and open Red Sea (light orange line) (Coello‐Camba and Agustí 2021). The orange line represents the relationship observed in this study.

*Synechococcus* abundance showed a negative relationship with temperature, as indicated by the significant linear regression (Figure 1a), which explained 22% of the variability (*p* < 0.0001, *N* = 20,706). Synechococcus abundance was not related to nutrients concentration and weakly positively correlated to salinity (Table S2).

As the relationship between SST and *Synechococcus* abundance was unimodal, we additionally fitted a Lorentzian curve, which revealed an increase in abundance with rising temperature, up to a critical point of 25.8°C ± 0.14°C (*p* < 0.0001, *R* ^2^ = 0.25; Figure 1a); beyond this, the relationship became negative. To better explore the upper temperature threshold, we focused in the maximum abundance of *Synechococcus* applying a Gaussian model to the absolute values of abundance (Figure 1b). This approach highlighted the symmetry of the maximum abundance distribution and indicated an optimum of 30.23°C; above this optimum maximum *Synechococcus* abundance decreased (Figure 1b). Together, these results indicated temperature limits to *Synechococcus* abundance well below the extreme temperatures recorded in recent years. Published data reported a positive relationship for subtropical and tropical oceans globally (Agustí et al. 2019). Other studies including both temperate (Stevens et al. 2023; Choi et al. 2013) and tropical waters (Chen et al. 2011) found that *Synechococcus* abundance followed a curve, showing a positive increase with temperature until reaching maximum abundance, after which abundance plateaued as temperature continued to rise (Figure 1c). In the open Red Sea, where temperatures reached up to 32.4°C, *Synechococcus* abundance increased to a critical point of 30.6°C before declining (Coello‐Camba and Agustí 2021). Our results showed a decline in *Synechococcus* abundance at SSTs exceeding previously observed temperature ranges (Figure 1c).

### 3.3 *Synechococcus* Thermal Tolerance

**FIGURE 2:**
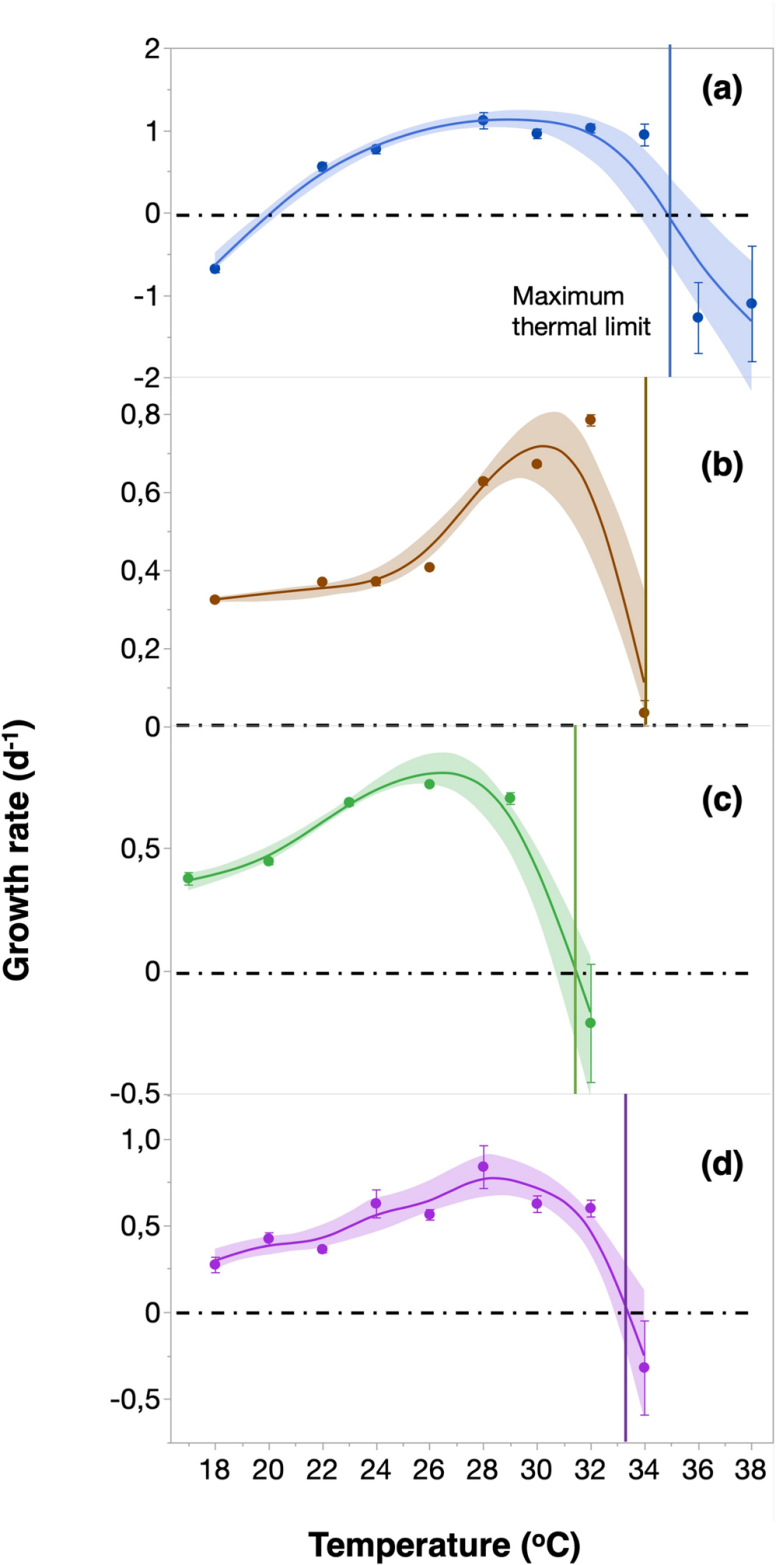
Thermal Performance of Red Sea *Synechococcus*. Average growth rates (day^−1^) of four Red Sea *Synechococcus* strains cultured at temperatures ranging from 17°C to 38°C. (a) Clade IIa, (b) Clade IX, (c), and (d) two strains of Clade IIIa. Error bars represent the standard error, and the blue, brown, green, and purple lines indicate the best‐fit curve, with shadow areas representing the 95% confidence interval. The dotted lines mark zero growth, and vertical color lines mark the maximum temperature for growth.

Recent warming may have exceeded the thermal limits of *Synechococcus*; therefore, we examined the thermal performance of several clades, including the dominant clade IIa. We grew clades IX and IIa, in addition to two strains of IIIa, in the laboratory, exposing them to a gradient of temperatures ranging from 17°C to 38°C. Thermal performance curves were constructed based on growth rates at each temperature, allowing us to determine the optimum growth temperature (*T*_opt_) and thermal limits for each strain (Figure 2). The optimum growth temperature (*T*_opt_) ranged from 25.41°C ± 1.03°C for clade IIIa to 33.16°C ± 0.23°C for clade IIa (Table S4). Clade IIa showed the highest maximum growth rate (1.04 day^−1^) and thermal limit (35.2°C) (Figure 2, Table S3). Although most Red Sea *Synechococcus* strains, particularly clade IIa, demonstrated considerable thermal tolerance, the maximum temperatures recorded at our sampling site during the warmest years exceeded the thermal limits of clade IIa and all other tested clades. Also, the differences in thermal performance between clades affected model performance: the lack of a distinct *T*_opt_ in Clade IIa reduces the accuracy of thermal performance curve fits, whereas the pronounced optimum of other clades, as Clade IX, allows a more robust parameterization.

### 3.4 Heatwave Events and *Synechococcus* Phenology

**FIGURE 3:**
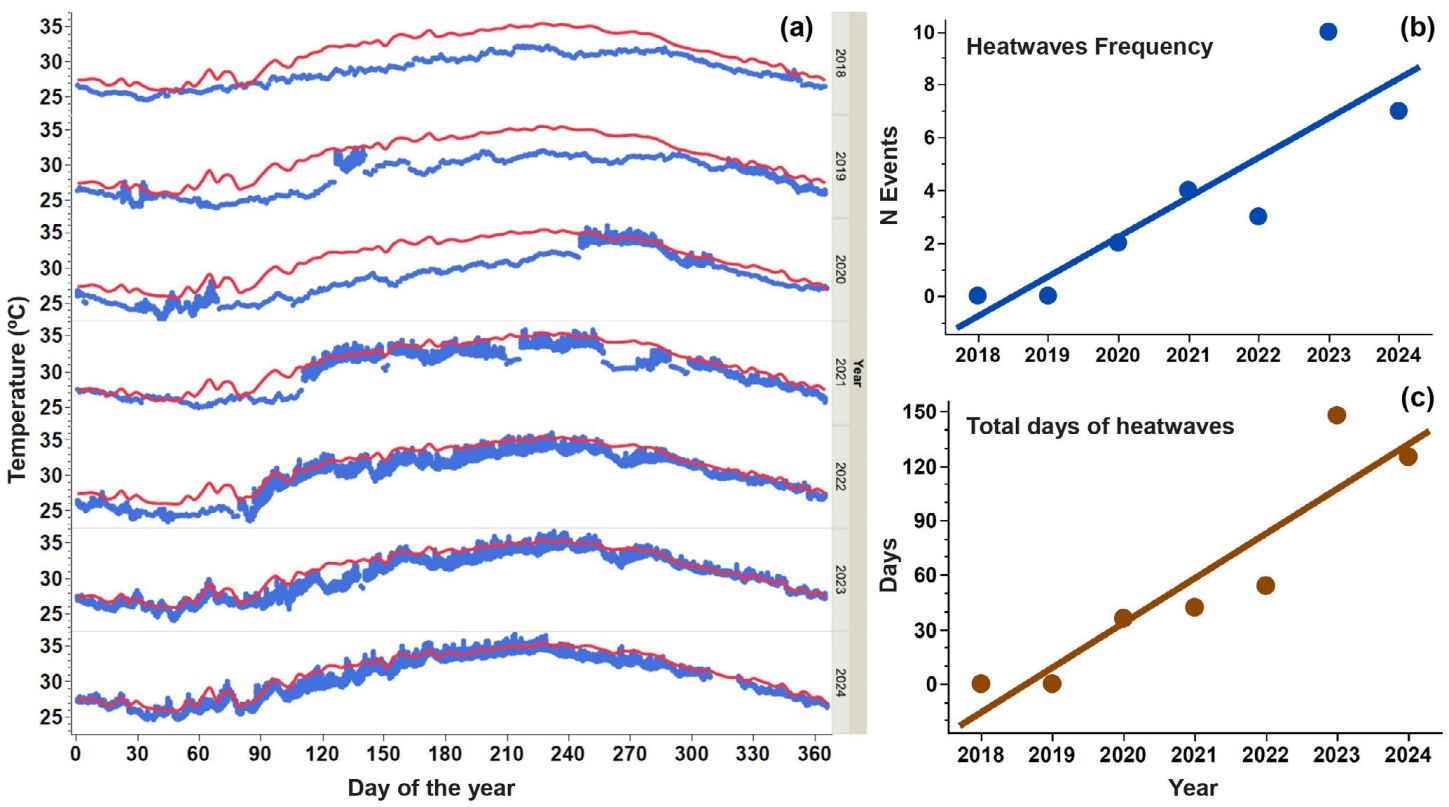
Marine Heatwave Events. (a) Daily temperature variation (blue dots) during the study period, with the 90th percentile of the 7‐year average temperature (red line) used to identify MHWs. (b) Linear increase in the frequency of MHW events over the years. (c) Linear increase in the duration (total days) of MHWs over the years.

No MHWs were observed in the first 2 years of the study; however, their frequency increased over time, peaking at 10 and 7 events in 2023 and 2024, respectively (Figure 3, Table S5). The duration of MHWs also increased, with a maximum of 55 consecutive days in the summer of 2024 (Figure 3).

**FIGURE 4:**
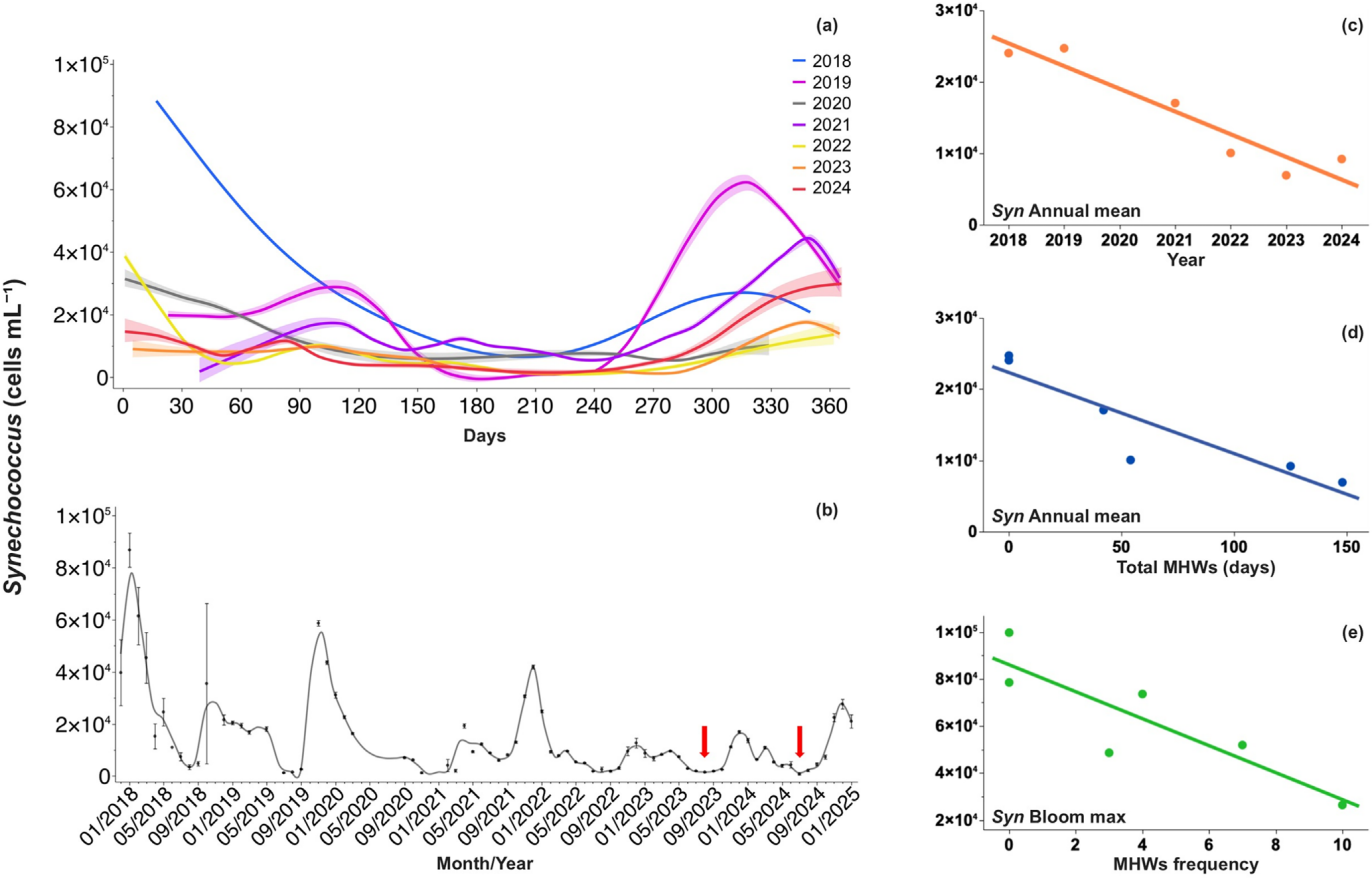
*Synechococcus* phenology and Marine Heatwave Events (MHWs). (a) Interannual variation in *Synechococcus* abundance, highlighting differences in bloom occurrence. (b) Monthly average (±SE) *Synechococcus* abundance throughout the study period. Red arrows indicate periods of lowest observed abundance. The line connecting the data points indicates the standard error. (c) Decline in annual mean *Synechococcus* abundance over time; the orange line represents the linear regression fit (*R* ^2^ = 0.85, *p* = 0.008). (d) Negative relationship between the total number of MHWs days per year and the annual mean *Synechococcus* abundance; blue line: Linear regression fit (*R* ^2^ = 0.88, *p* = 0.005). (e) Negative relationship between annual MHW frequency and the maximum *Synechococcus* abundance during the largest bloom; green line: Linear regression fit (*R* ^2^ = 0.79, *p* = 0.0017).

There was a clear upward trend in the total number of MHW days over the study period, with 148 and 125 days in 2023 and 2024, respectively (Figure 3b,c). This increase in heatwave events and duration had a clear effect on *Synechococcus* phenology. Typically, a large bloom occurs in late fall, extending into winter, with an average duration of 13 ± 1 weeks (Figure 4a,b). However, the magnitude of the annual bloom eventually decreased by 4.5 times in the warmest years (Figure 4b). The lowest abundance was consistently observed in summer, with especially low values of 85 and 71 cells mL^−1^ in 2023 and 2024, respectively (Figure 4b, Table 1). No significant changes were observed in the timing of bloom initiation or termination. Annual mean *Synechococcus* abundance declined over time, with peak abundance in 2018 and 2019 (Figure 4c), years that lacked the extreme thermal stress observed when temperatures exceeded 36°C. The increasing thermal stress from more frequent and intense MHWs is reflected in the linear decline of mean annual *Synechococcus* abundance with increasing number of heatwaves days (Figure 3d), as well as in the progressive reduction of *Synechococcus* bloom magnitude over time as MHW frequency increased (Figure 3e). The major bloom typically began as temperatures dropped following the hot summer months, usually around week 43 (October) (Table S6). The rate of temperature decline from peak summer values to bloom initiation was faster in the warmest years (2023–2024) than in the coolest years (2018 and 2019), with dissipation rates of 0.25°C and 0.18°C month^−1^ for the cool years and 1.29°C and 1.19°C month^−1^ for the warmest years (Table S5). Despite these changes, the growth rate during the bloom period remained steady across years at an average of 0.015 ± 0.0008 day^−1^, showing no significant differences. Notably, the decline in bloom magnitude was not influenced by changes in this growth rate but by lower *Synechococcus* abundances at bloom initiation in the warmest year.

## 4 Discussion

Our study reveals a significant decline in *Synechococcus* abundance in recent years, particularly in 2023–2024, when SST exceeded the region's previously recorded maximum temperature. This unprecedented warming resulted in an increased frequency and prolonged duration of MHWs, substantially affecting *Synechococcus* populations. Our data suggest that *Synechococcus* surpassed its thermal threshold, effectively losing its thermal niche in the environment. Although the large‐scale impacts of warming and heatwaves on marine organisms such as corals, seagrasses, and fish stocks are well‐documented (Cheung et al. 2021; de Luzinais et al. 2024; Hughes et al. 2017; Marbà et al. 2022; Nguyen et al. 2021; Pörtner and Peck 2010), our study reported the decline of a marine picophytoplankton population—a crucial component of oligotrophic oceans—due to extreme ocean warming.

As utilized in our study, high‐frequency sampling was crucial for capturing the rapid onset and progression of population shifts in response to temperature changes. This high temporal resolution provided critical insights into transient ecological responses and heat waves (Schaeffer and Roughan 2017). Our data suggest that temperature acts as a physiological constraint that emerges near thermal extremes, rather than as a dominant predictor across the full temperature range. This pattern is expected for in situ plankton populations which are influenced by multiple interacting drivers, including light availability, nutrient supply, grazing, and viral lysis (Agawin et al. 1998; Li 1998; Danovaro et al. 2011). Consistent with this interpretation, the relatively low *R* ^2^ values observed here can be reconciled with the marked population decline under extreme warming. Previous field‐based studies have similarly reported modest explanatory power of temperature for *Synechococcus* abundance, despite clear temperature‐related patterns and identifiable thermal thresholds (Morán et al. 2010; Stevens et al. 2023). Previous studies on *Synechococcus* abundance and temperature did not include temperatures above 30°C, and several studies from temperate waters (0°C–30°C; Morán et al. 2010; Chang et al. 1996; Wang et al. 2011) typically showed a linear increase in *Synechococcus* abundance with temperature within the temperature range of each study area.

Our findings indicate that *Synechococcus* populations decline when SSTs exceed their thermal optimum, as extreme warming restricts the persistence of standing strain‐level diversity, despite continued growth of a subset of thermally tolerant strains. Previous studies have documented the genetic diversity of Red Sea *Synechococcus* (Coello‐Camba and Agustí 2024; Farrant et al. 2016), revealing that clade IIa dominates the population, although other clades (CRD1, EnvA, II, III, VII, IX, and WPC1) and subclades (IIb, IIc, IIe, IIf, IIIa, and IIIb) are also present. *Synechococcus* populations in the Red Sea are therefore expected to exhibit high thermal tolerance. Consistent with this, Clade IIa constitutes > 85% of the Red Sea picophytoplankton when seawater temperatures exceed 30°C (Coello‐Camba and Agustí 2024), highlighting its high thermal tolerance. Our experimental thermal performance results further confirm the high thermal tolerance of Clade IIa. The contrasting thermal responses among clades highlight distinct ecological strategies within *Synechococcus*. Clade IIa exhibits a generalist thermal strategy, maintaining high growth rates across a broad temperature range without a clearly defined thermal optimum (Angilletta 2009). This strategy likely confers resilience under thermally variable or progressively warming conditions, such as those characteristic of the Red Sea. In contrast, clade IX displays a specialist thermal response, with high growth near its thermal optimum followed by a sharp decline beyond this threshold (Angilletta 2009). While such specialization may maximize performance within a narrow thermal window, it also increases vulnerability when environmental temperatures exceed physiological limits.

A MHW is a period of extremely high seawater temperatures that persist for several days relative to the climatological averages and can have devastating impacts on marine ecosystems. *Synechococcus* blooms occurred under more favorable temperatures, while minimum abundance coincided with peak annual SST. The magnitude of the largest annual bloom declined over time, decreasing by up to 4.5‐fold in the recent warmest years. This pattern correlated with the increasing frequency of MHW events, which rose from none at the beginning of the study to more than seven in the most extreme warming years. Annual *Synechococcus* abundance, integrating both maximum and minimum values, declined in recent years and showed a close association with the increase in the total number of MHW days. Although the observation period was limited and precludes robust statistical conclusions, our MHW observations captured periods of accelerated warming, and are consistent with numerous impact studies that necessarily examine discrete extreme events over constrained time windows (e.g., Wernberg et al. 2012; Arias‐Ortiz et al. 2018; Peña et al. 2019). Previous studies on phytoplankton phenology suggest that such shifts typically require at least 10 years to become significant (Record et al. 2019).

Beyond exceeding *Synechococcus* thermal limits, warming may also influence other ecological factors, contributing to population collapse. The Red Sea is an oligotrophic basin characterized by the absence of riverine inputs, where nutrients availability modulates the photosynthetic performance and growth of phytoplankton communities across the basin (López‐Sandoval et al. 2021). Besides, nutrient availability may interact with thermal stress to influence population dynamics, this study was conducted in a pristine coastal area far from populated and industrial regions. Inorganic nutrient concentrations remained low throughout the study period and comparison with previous measurements in the same coastal site (López‐Sandoval et al. 2021) indicated no significant changes in nutrient concentration. The collapse of experimentally induced *Synechococcus* blooms by nutrients additions in the Red Sea due to cyanophage infections has been documented (Coello‐Camba et al. 2020); however, the effects of increasing seawater temperatures on cyanophage dynamics remain unclear (Chu et al. 2011; Danovaro et al. 2011). Viral infections typically depend on increased host abundance, which, for Red Sea *Synechococcus*, occurs when temperatures decline.

Although grazing could potentially contribute to the observed Synechococcus decline, microzooplankton dynamics were not measured in this study, and regional data on grazing processes in the Red Sea remain scarce. Previous results from the coastal Red Sea provided little evidence that warming enhances top‐down control on *Synechococcus*, as heterotrophic nanoflagellates primarily regulate heterotrophic bacteria rather than picocyanobacteria (Sabbagh et al. 2020). Shipboard dilution experiments in the oligotrophic Gulf of Aqaba and northern Red Sea indicated strong prey selectivity as although strong grazing on heterotrophic bacteria and eukaryotic phytoplankton was reported, very low or undetectable grazing on *Synechococcus* was measured (Sommer et al. 2002). Complementary dilution experiments in the Red Sea further showed that *Synechococcus* was neither strongly nutrient‐limited nor substantially controlled by grazing, while microbial regulation primarily targeted bacteria and small eukaryotic autotrophs (Berninger and Wickham 2005).

While it is expected that warming may affect grazing pressure, its direction and magnitude remain uncertain (Rohr et al. 2023). The Metabolic Theory of Ecology (MTE) predicts that heterotrophic processes, including mortality and grazing, exhibit higher thermal sensitivity than autotrophic processes, leading to the expectation of enhanced grazing pressure under warming (Brown et al. 2004; López‐Urrutia et al. 2006; Rose and Caron 2007; O'Connor et al. 2009). This framework has been challenged, as autotrophic processes can be equally or more temperature‐sensitive, and grazing responses depend strongly on nutrient conditions (Chen and Laws 2017; Hayashida et al. 2020). Under extreme warming, phytoplankton growth may temporarily outpace grazing, weakening top‐down control (Cabrerizo et al. 2024). Grazing–temperature relationships have rarely been tested for picocyanobacteria such as *Synechococcus*, particularly in oligotrophic tropical systems.

As warming reduces *Synechococcus* populations, ecological opportunities emerge for more heat‐tolerant, potentially harmful taxa. Research in the Red Sea indicates that while general diversity declines under extreme heat, specific taxa thrive as identified for *Protoperidinium quinquecorne* for which documented blooms are reported at temperatures reaching 34°C (Alkawri, Abker, et al. 2016). Also, the toxic *Pyrodinium bahamense* showed peaks during the hottest months (June–August) at temperatures up to 32°C (Alkawri, Areeki, and Alsharaby 2016; Banguera‐Hinestroza et al. 2016). Ultimately, rising sea surface temperatures expand the “window of opportunity” for these resilient, harmful taxa to outcompete more sensitive species (Mohamed 2018; Prabowo and Agusti 2019).

The unprecedented warming reported herein was observed globally in 2023–2024, with mean temperatures exceeding 1.5°C above preindustrial levels (Ripple et al. 2024). While the IPCC projected that this threshold would be reached in the early 2030s, it was already exceeded in 2024. This extreme warming has contributed to widespread coral bleaching in tropical oceans (Goreau and Hayes 2024), unprecedented wildfires in Canada (Jain et al. 2024), and exceptionally low sea ice extent in the Arctic and Antarctica (Davies et al. 2024; Moon et al. 2024; Purich and Doddridge 2023). In the tropical ocean, these conditions represent a new thermal niche. Tropical organisms are considered particularly vulnerable to warming because they already live near their physiological limits (Deutsch et al. 2008; Thomas et al. 2012). In the absence of an evolutionary response, organisms must migrate poleward to survive (Thomas et al. 2012). Although *Synechococcus* could evolve rapidly, evolutionary rescue under extreme warming is not guaranteed when environmental change outpaces adaptation, potentially causing population decline despite ongoing selection (Chevin et al. 2013). Under such conditions, strain sorting and selective persistence of pre‐adapted lineages are more likely than rapid evolutionary increases in thermal tolerance (Reusch 2014). *Synechococcus* faces these challenges, raising concerns about the future of this population and its ecosystems. The decline of *Synechococcus* may allow other phytoplankton—including potentially harmful algal species—to occupy the open niche, with cascading effects on higher trophic levels. Our study provides critical insights into the impacts of extreme warming on photosynthetic plankton populations and oceanic primary production.

## Author Contributions

**Luthfiyyah Azizah:** methodology, investigation, visualization, writing original draft, review and editing. **Eva Alou‐Font:** methodology, investigation, visualization, project administration, writing original draft, review and editing. **Alexandra Coello‐Camba:** methodology, investigation, visualization, writing original draft, review. **Susana Agusti:** conceptualization, methodology, investigation, visualization, funding acquisition, project administration, supervision, writing original draft, review and editing.

## Funding

This research was supported by the King Abdullah University of Science and Technology (KAUST).

## Conflicts of Interest

The authors declare no conflicts of interest.

## Supporting information


**Data S1:** gcb70791‐sup‐0001‐Supinfo.docx.

## Data Availability

All data supporting this study are available in the KAUST repository at https://doi.org/10.25781/KAUST‐O84M5. Sea surface temperature data from Copernicus is available https://doi.org/10.48670/moi‐00168.
