# Supplementary material for: Thermal Limits and Decline of Synechococcus Under Accelerated Warming and Marine Heatwaves
Source: Glob Chang Biol. 2026 Mar 11;32(3):e70791. doi: 10.1111/gcb.70791 (PMC12976987; doi:10.1111/gcb.70791)
Supplement: Supplementary file 1 — Data S1: gcb70791‐sup‐0001‐Supinfo.docx. [file GCB-32-e70791-s001.docx]

Supplementary Materials for

**Thermal Limits and Decline of *Synechococcus* under Accelerated Warming and Marine Heatwaves**

**Luthfiyyah Azizah^1^, Eva Alou-Font^1^, Alexandra Coello-Camba^2^, Susana Agusti^1*^**

^1^King Abdullah University of Science and Technology (KAUST), Biological and Environmental Science and Engineering Division, Thuwal, Saudi Arabia

^2^Universidad Internacional de Valencia (VIU), Calle Pintor Sorolla 21, 46002, Valencia, Spain

*Corresponding author email: [susana.agusti@kaust.edu.sa](mailto:susana.agusti@kaust.edu.sa)

The PDF file includes:

Figures S1 to S4

Tables S1 to S6

**Supplementary Figures**


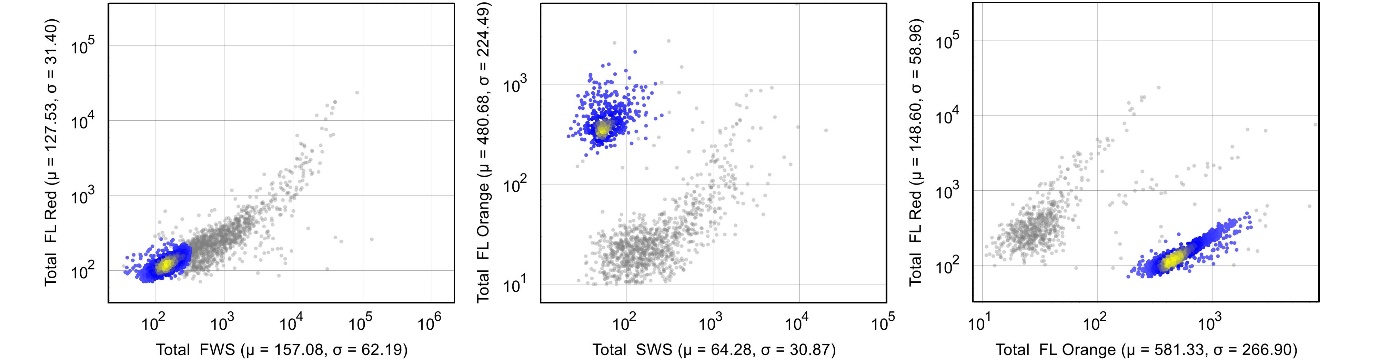


**Figure S1 | Cytogram to identified *Synechococcus* population. A)** Total fluorescence red vs. total forward scattered. **B)** Total fluorescence orange vs. total sideward scattered. **C)** Total fluoresncence red vs. total fluorescence orange (blue dots identified *Synechococcus* population).


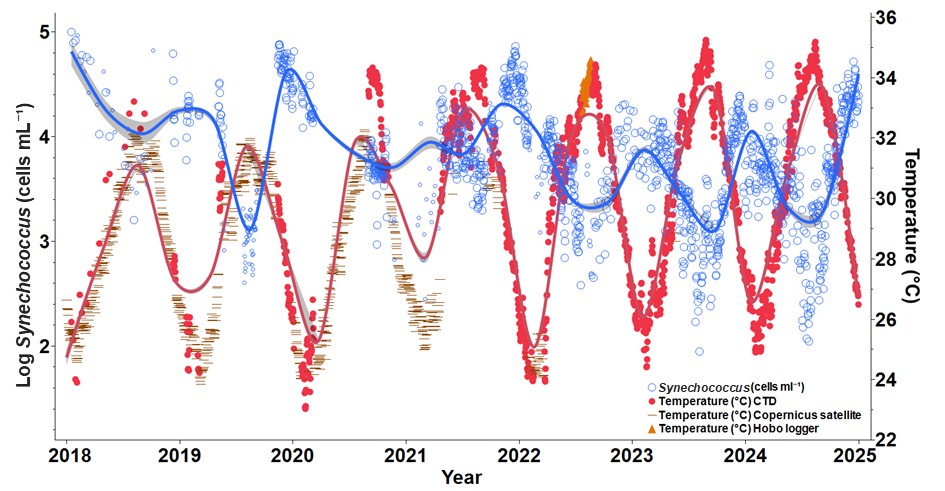


**Figure S2 | Temporal changes in *Synechococcus* abundance and temperature (2018–2024).** Mean daily Synechococcus abundance (blue circles) and temperature from 2018–2024. Temperature data include Copernicus satellite SST (brown stars, N = 1,031), in situ CTD measurements (red circles, N = 34,027), and HOBO loggers (orange triangles, N = 731). Solid lines represent smoothing spline fits (λ = 0.05), with shaded bands indicating 95% confidence intervals.

**Figure S3 | Inorganic nutrient concentrations.** Concentrations (μmol L^−1^) of (**A**) silicate, (**B**) nitrate, and (**C**) phosphate in the coastal waters during 2021–2023, and the first 5 months of 2024. Smooth lines indicate fitted trends.


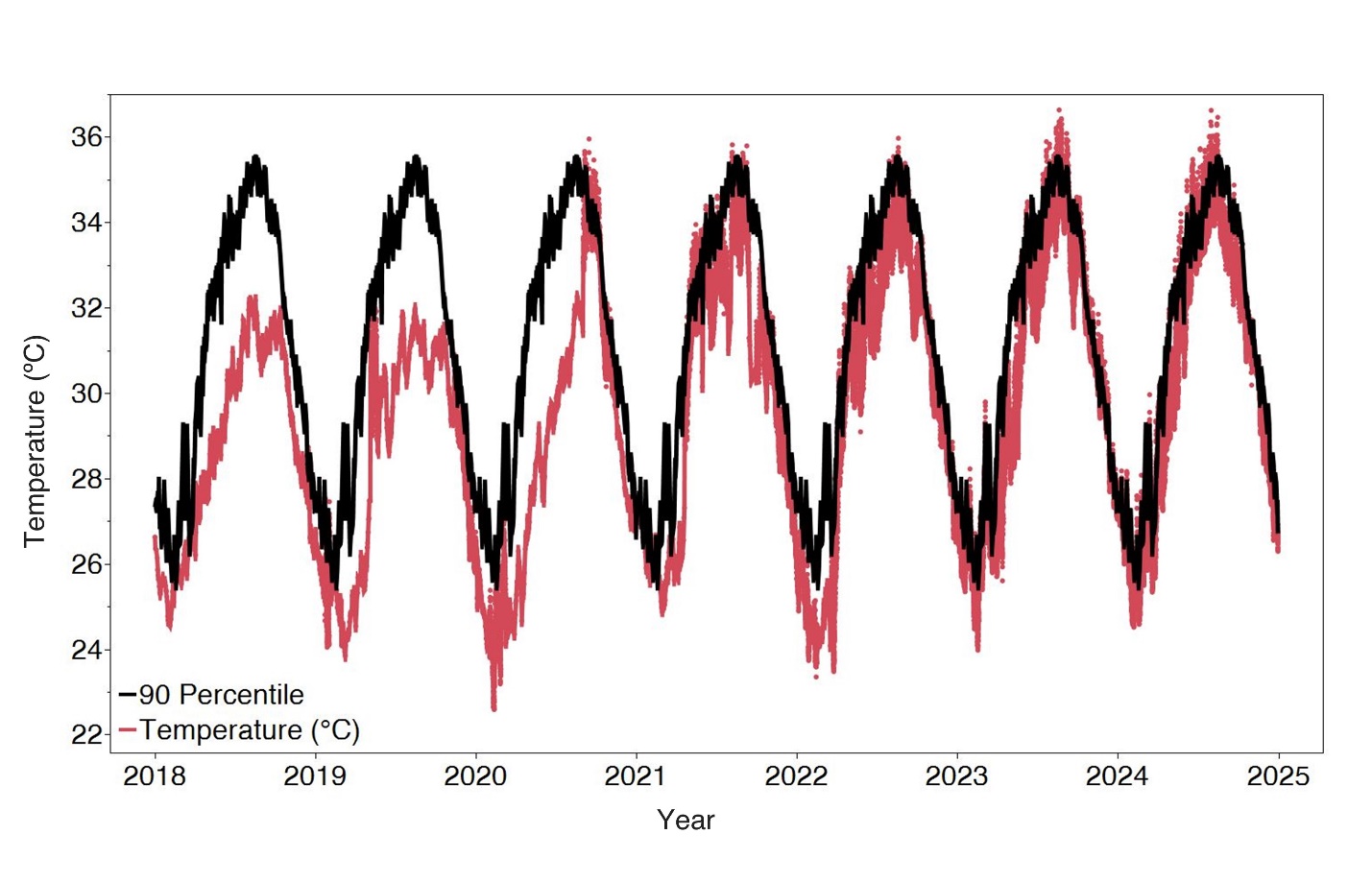


**Figure S4 |** **Marine heatwave events.** Daily sea surface temperature (SST) observations in the coastal Red Sea (red dots) overlaid with the 90^th^ percentile threshold (black line) based on the 7-year daily average. The SST data from January 2018 to April 2021 were obtained from satellite observations (Copernicus), whereas data from May 2021 to December 2024 were obtained from deployed CTD units.

**Table S1 | Annual average nutrient concentrations (μmol L^−1^) (± Standard Error, SE) in the coastal Red Sea sampling site in 2021–2023 and the first 5 months of 2024^+^.** Data from 2017 *and 2018* correspond to a close sampling site in the same coastal area (Lopez-Sandoval et al. 2021). The analyzed nutrients include NO_3_^−^ , PO_4_^3−^, and SiO_2_^4−^. No significant differences were observed between years (ANOVA, p < 0.05).

| **Year** | **N** | **NO_3_^−^** **± SE** | **PO_4_^3−^ ± SE** | **SiO_2_ ± SE** |
| --- | --- | --- | --- | --- |
| *2017 | 24 | 0.85±0.17 | 0.14±0.004 | 0.76±0.04 |
| *2018 | 20 | 0.70±0.13 | 0.11±0.02 | 0.71±0.30 |
| 2021 | 23 | 0.99 ± 0.14 | 0.07 ± 0.01 | 1.11 ± 0.07 |
| 2022 | 22 | 0.87 ± 0.20 | 0.12 ± 0.03 | 1.16 ± 0.11 |
| 2023 | 21 | 1.01 ± 0.17 | 0.15 ± 0.05 | 1.22 ± 0.13 |
| 2024 | 10 | 0.47 ± 0.10 | 0.03 ± 0.01 | 0.93 ± 0.02 |

López‐Sandoval, D. C., Duarte, C. M., & Agustí, S. (2021). Nutrient and temperature constraints on primary production and net phytoplankton growth in a tropical ecosystem. *Limnology and Oceanography*, *66*(7), 2923-2935.

**Table S2 | Summary of linear regression statistics for *Synechococcus* and environmental parameters relationships.** Linear regression analyses reporting intercept ± standard error (SE), slope ± SE, p-value, coefficient of determination (R²), sample size (N), and root mean square error (RMSE) for relationships between *Synechococcus* abundance metrics, environmental variables, and marine heatwave (MHW) characteristics, using the same data and transformations as in the corresponding figures. Asterisks denote *P* < 0.05.

|  | *Intercept* ± SE | *Slope* ± SE | | p | R^2^ | N | RMSE |  |
| --- | --- | --- | --- | --- | --- | --- | --- | --- |
| *Synechococcus* log_10_ (cells mL^-1^) vs: | | | | | | | | |
| Temperature (^o^C) | 6.53 ± 0.03 | -0.089 ± 0.001 | | < 0.0001* | 0.22 | 20706 | 0.48 |  |
| Salinity (psu) | 14.05 ± 0.41 | -0.26 ± 0.01 | | < 0.0001* | 0.036 | 1652 | 0.52 |  |
| Phosphate log_10_ (µM) | 3.63 ± 0.34 | -0.023 ± 0.11 | | 0.83 | <0.001 | 62 | 0.48 |  |
| Nitrate log_10_ (µM) | 3.71 ± 0.15 | 0.041 ± 0.15 | | 0.78 | <0.001 | 74 | 0.49 |  |
| MHWs frequency (dependent) vs: | | | | | | | | |
| Year | −−3027 ± 733 | 1.5 ± 0.36 | | 0.009* | 0.77 | 7 | 1.92 |  |
| Total MHWs (days) vs | | | | | | | | |
| Year | −49673± 9538 | | 24.61 ± 4.72 | 0.003* | 0.84 | 7 | 24.97 |  |
| Mean *Synechococcus* log_10_ (cells mL⁻¹) vs: | | | | | | | | |
| Year | 4.43 ± 0.07 | -0.09 ± 0.02 | | 0.008* | 0.85 | 6 | 0.09 |  |
| Total MHWs (days) | 4.35 ± 0.05 | -0.003 ± 0.0006 | | 0.005* | 0.88 | 6 | 0.08 |  |
| *Synechococcus* log_10_ (cells mL⁻¹) at bloom maximum vs: | | | | | | | | |
| MHWs frequency | 4.95 ± 0.06 | -0.04 ± 0.01 | | 0.017* | 0.79 | 6 | 0.104 |  |

**Table S3 | Summary of reported relationships between temperature and *Synechococcus* abundance across various marine regions.** The table includes the temperature range (°C), minimum and maximum temperature values, and the number of observations (n) from each study.

| **Relationship** | **Temperature (**°**C)** | | | **N** | **Regions** | | **Location** | | **Reference** | |
| --- | --- | --- | --- | --- | --- | --- | --- | --- | --- | --- |
|  | **Minimum** | **Maximum** | **Range** |  | |  | |  | |  |
| Linear | 1.7 | 29.4 | 27.7 | 213 | | Temperate | | Chesapeake  Bay | | (48) |
| Lorentzian | 5 | 25 | 20 | 61,113 | | Temperate | | Northeastern Atlantic | | (34) |
| Linear | 0.6 | 22 | 21.4 | 59 | | Temperate | | Northwestern Atlantic | | (20) |
| Linear | 9.1 | 30 | 20.9 | 36 | | Temperate | | Sea of Japan | | (49) |
| Lorentzian | 16.2 | 30.5 | 14.3 | 135 | | Subtropical | | South China Sea | | (50) |
| Linear | 17 | 30 | 13 | 66 | | Subtropical | | Coast of Taiwan | | (4*7*) |
| Linear | 12.4 | 29.3 | 16.9 | 1,077 | | Subtropical–Tropical | | Atlantic, Indian, Pacific | | (32) |
| Lorentzian | 21.4 | 32.4 | 11 | 161 | | Tropical | | Red Sea (open waters) | | (41) |
| Lorentzian | 22.6 | 36.6 | 14 | 20,648 | | Tropical | | Red Sea | | This study |

**Table S4 | Thermal parameters of different Red Sea *Synechococcus* strains.** This table includes optimum growth temperatures (*T_opt_*) and maximum growth rates (*µ_max_*) estimated using the Dell model. Minimum (*T_min_*) and maximum temperatures (*T_max_*), along with the thermal range (*T_max_* – *T_min_*, range), were determined using a linear model.

| **(Sub)clade** | ***T_opt_* ± SE** | ***µ_max_* ± SE** | **Temperature (**°**C)** | | |
| --- | --- | --- | --- | --- | --- |
|  |  |  | **Minimum** | **Maximum** | **Range** |
| IIIa (2529) | 31.10 ± 0.20 | 0.65 ± 0.02 | 10.8 | 33.0 | 22.2 |
| IIa | 33.16 ± 0.23 | 1.04 ± 0.18 | 11.4 | 35.2 | 23.8 |
| IX | 31.71 ± 0.34 | 0.65 ± 0.02 | 13.7 | 34.1 | 19.9 |
| IIIa (2553) | 25.41 ± 1.03 | 0.78 ± 0.06 | 14.0 | 31.5 | 17.5 |

**Table S5 | Interannual marine heatwave events (MHWs) detected over the 7-year study period.** This table includes the frequency of MHW events (number of events per year), maximum duration of a single MHW event (number of days), total number of MHW days per year (sum of event days), and monthly heat dissipation rate (calculated as the slope between the maximum temperature in summer and the temperature at bloom initiation).

| **Year** | **Frequency (N)** | **Max event duration (days)** | **Total days (days)** | **Heat dissipation rate (**°**C month^−1^)** |
| --- | --- | --- | --- | --- |
| 2018 | 0 | 0 | 0 | 0.25 |
| 2019 | 0 | 0 | 0 | 0.18 |
| 2020 | 2 | 29 | 36 | - |
| 2021 | 4 | 20 | 42 | 1.2 |
| 2022 | 3 | 31 | 54 | 0.89 |
| 2023 | 10 | 29 | 148 | 0.97 |
| 2024 | 7 | 55 | 125 | 1.3 |

**Table S6 | *Synechococcus* phenology and temperature during the bloom period over the 7-year study.** The table includes the week of bloom initiation (*Bi*), *Synechococcus* abundance during the bloom period (*Syn*), reported as mean and maximum abundance (10^3^ cells mL^−1^), and the growth rate (µ) during the bloom (calculated as the slope between the day of bloom initiation (*Bi*) and the peak bloom (d^−1^)). Additionally, the temperature at bloom initiation (T*_Bi_*) and the mean temperature throughout the bloom period are shown.

| **Year** | ***Bi* (week)** | ***Syn* (10^3^ cells mL^−1^)** | | **µ (d^−1^)** | **Temperature (**°**C)** | |
| --- | --- | --- | --- | --- | --- | --- |
|  |  | **Mean ± SE** | **Max** |  | **T*_Bi_*** | **Mean** |
| 2018 | 45* | - | 99.7 | 0.013 | 31.39 | 27.86 ± 0.06 |
| 2019 | 46* | 47.6 ± 2.09 | 78.5 | 0.017 | 30.27 | 27.99 ± 0.19 |
| 2020 | - | - | - | - | - | - |
| 2021 | 42 | 37.0 ± 2.12 | 73.6 | 0.016 | 32.09 | 28.59 ± 0.16 |
| 2022 | 44 | 9.44 ± 1.02 | 48.6 | 0.018 | 32.18 | 28.56 ± 0.18 |
| 2023 | 43 | 12.2 ± 0.72 | 26.4 | 0.015 | 32.01 | 29.44 ± 0.03 |
| 2024 | 42 | 16.5 ± 1.03 | 51.9 | 0.012 | 32.10 | 30.64 ± 0.05 |

*Initiation of bloom was interpolated from the lowest fit due to data gaps.
